# Spider-Venom Peptides as Therapeutics

**DOI:** 10.3390/toxins2122851

**Published:** 2010-12-20

**Authors:** Natalie J. Saez, Sebastian Senff, Jonas E. Jensen, Sing Yan Er, Volker Herzig, Lachlan D. Rash, Glenn F. King

**Affiliations:** Institute for Molecular Bioscience, The University of Queensland, St Lucia, Queensland, 4072, Australia; Email: n.saez@imb.uq.edu.au (N.J.S.); s.senff@imb.uq.edu.au (S.S.); j.jensen@imb.uq.edu.au (J.E.J.); s.er@imb.uq.edu.au (S.Y.E.); v.herzig@imb.uq.edu.au (V.H.); l.rash@imb.uq.edu.au (L.D.R.)

**Keywords:** spider venom, peptide, therapeutics, drugs, drug discovery, cystine knot

## Abstract

Spiders are the most successful venomous animals and the most abundant terrestrial predators. Their remarkable success is due in large part to their ingenious exploitation of silk and the evolution of pharmacologically complex venoms that ensure rapid subjugation of prey. Most spider venoms are dominated by disulfide-rich peptides that typically have high affinity and specificity for particular subtypes of ion channels and receptors. Spider venoms are conservatively predicted to contain more than 10 million bioactive peptides, making them a valuable resource for drug discovery. Here we review the structure and pharmacology of spider-venom peptides that are being used as leads for the development of therapeutics against a wide range of pathophysiological conditions including cardiovascular disorders, chronic pain, inflammation, and erectile dysfunction.

## 1. Introduction: The Diverse Pharmacology of Spider Venoms

Spiders are the most successful venomous animals with an estimated 100,000 extant species [1]. The vast majority of spiders employ a lethal cocktail to rapidly subdue their prey, which are often many times their own size. However, despite their fearsome reputation, less than a handful of these insect assassins are harmful to humans [2,3]. Nevertheless, it is this small group of medically important species that first prompted scientists more than half a century ago to begin exploring the remarkable pharmacological diversity of spider venoms.

Amongst the ranks of animals that employ venom for their survival, spiders are the most successful, the most geographically widespread, and arguably consume the most diverse range of prey. Although the predominant items on a spider’s dinner menu are other arthropods, larger species will readily kill and feed on small fish, reptiles, amphibians, birds, and mammals. Thus, spider venoms contain a wealth of toxins that target a diverse range of receptors, channels, and enzymes in a wide range of vertebrate and invertebrate species.

Spider venoms are complex cocktails composed of a variety of compounds, including salts, small organic molecules, peptides, and proteins [4,5,6,7,8,9]. However, peptides are the primary components of spider venoms, and some species produce venom containing >1000 unique peptides of mass 2–8 kDa [10]. Based on the number of described spider species and a relatively conservative estimate of the complexity of their venom it has been estimated that the potential number of unique spider venom peptides could be upwards of 12 million [11]. In recent years there has been an exponential increase in the number of spider-toxin sequences being reported [12] due to the application of high-throughput proteomic [13,14] and transcriptomic [15,16,17] approaches, or a combination of these methods [10,18,19]. In the last 18 months alone the number of toxins in the ArachnoServer spider-toxin database [20,21] has more than doubled, and is now excess of 900 (see http://www.arachnoserver.org/). Nevertheless, our knowledge of the diversity of spider-venom peptides is still rudimentary, with less than 0.01% of potential peptides having been isolated and studied.

Although only a small number of spider venom peptides have been pharmacologically characterized, the array of known biological activities is impressive [9]. In addition to the well known neurotoxic effects of spider venoms, they contain peptides with antiarrhythmic, antimicrobial, analgesic, antiparasitic, cytolytic, haemolytic, and enzyme inhibitory activity. Furthermore, the crude venom of *Macrothele raveni* has antitumor activity, for which the responsible component has not yet been identified [22,23]. Finally, larger toxins such as the latrotoxins from the infamous black widow spider (*Latrodectus mactans*) and related species induce neurotransmitter release and they have played an important role in dissecting the process of synaptic vesicle exocytosis [24].

Since spiders employ their venom primarily to paralyse prey, it is no surprise that these venoms contain an abundance of peptides that modulate the activity of neuronal ion channels and receptors. Indeed, the majority of characterized spider-venom peptides target voltage-gated potassium (K_V_) [25], calcium (Ca_V_) [26,27], or sodium (Na_V_) [26,28] channels. More recently, novel spider-venom peptides have been found that interact with ligand-gated channels (e.g., purinergic receptors [29]) and recently discovered families of channels such as acid sensing ion channels [30], mechanosensitive channels [31], and transient receptor potential channels [32]. Not only do most of these peptides have selectivity for a given *class* of ion channel, they can have anything from mild preference to exquisite selectivity for a given channel *subtype*. This potential for high target affinity and selectivity makes spider-venom peptides an ideal natural source for the discovery of novel therapeutic leads [33].

Despite the advent of automation and the rise of high-throughput and high-content screening in the pharmaceutical industry there has been a sharp decline in the rate of discovery and development of novel chemical entities [34,35]. We recently reviewed the emerging role that venom-derived components can play in addressing this decline with an emphasis on technical advances that can aid the discovery process [36]. It is worth noting that, as of 2008, two of the 20 FDA-approved peptide pharmaceuticals were derived from animal venoms (*i.e.*, ziconitide and exendin-4) [37]. In this review we specifically examine the structure, targets, and mechanisms of action of spider-venom peptides with potential therapeutic applications.

## 2. Peptide Nomenclature

All peptide names are based on the rational nomenclature for naming peptide toxins that has been adopted by ArachnoServer and UniProtKB [12]. Common synonyms are also provided.

## 3. The Magical Properties of the Inhibitor Cystine Knot

**Figure 1:**
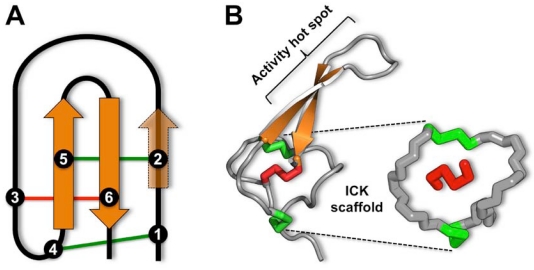
(A) The inhibitor cystine knot (ICK) motif comprises an antiparallel β sheet stabilized by a cystine knot. β strands are shown in orange and the six cysteine residues that form the cystine knot are labeled 1–6. In spider toxins, the β sheet typically comprises only the two β strands housing cysteine residues 5 and 6, although a third N-terminal strand encompassing cysteine 2 is sometimes present. The two “outer” disulfide bonds are shown in green and the “inner” disulfide bridge is red. (B) The cystine knot of the 37-residue spider-venom peptide ω-hexatoxin-Hv1a [43].The cystine knot comprises a ring formed by two disulfides (green) and the intervening sections of polypeptide backbone (gray), with a third disulfide (red) piercing the ring to create a pseudo-knot. The hydrophobic core of the toxin consists primarily of the two central disulfide bridges connected to the β strands. Key functional residues in ICK toxins are often located in the β hairpin that projects from the central disulfide-rich core of the peptide.

Peptides have generally been considered poor candidates for human therapeutics because of their susceptibility to proteolytic degradation *in vivo* and their limited penetration of intestinal mucosa [37,38]. However, in contrast with most peptides, the presence of an inhibitor cystine knot (ICK) in most spider-venom toxins provides these peptides with extraordinary stability. The inhibitor cystine knot (ICK) is defined as an antiparallel β sheet stabilized by a cystine knot [39,40,41]. In spider toxins, the β sheet typically comprises only two β strands although a third N-terminal strand is sometimes present (Figure 1A) [42]. The cystine knot comprises a ring formed by two disulfides and the intervening sections of polypeptide backbone, with a third disulfide piercing the ring to create a pseudo-knot (Figure 1B). The compact hydrophobic core of the ICK motif consists primarily of the two central disulfide bridges that emanate from the two β strands that characterize the ICK fold [43]. Except for the special case of cyclic ICK peptides, cystine knots are not true knots in the mathematical sense as they can be untied by a non-bond-breaking geometrical transformation [44]. Nevertheless, the cystine knot converts ICK toxins into hyperstable mini-proteins with tremendous chemical, thermal, and biological stability. ICK toxins are typically resistant to extremes of pH, organic solvents, and high temperatures [45]. However, from a therapeutic perspective, their most important property is their resistance to proteases; ICK peptides are typically stable in human serum for several days and have half-lives in simulated gastric fluid [46] of >12 hours (GFK and VH, unpublished). It was recently demonstrated that stabilization of a 16-residue α-conotoxin through cyclization dramatically increased its oral activity [47], and it is therefore possible that the inherent stability of ICK peptides might impart them with oral activity without the need to introduce exotic modifications.

ICK toxins have proliferated in spider venoms to the point where they now dominate most spider-venom peptidomes. The marked insensitivity of this structural scaffold to changes in intercystine residues has enabled spiders to develop diverse pharmacologies using the same disulfide framework [48]. Moreover, many of these ICK peptides not only have high affinity but also exquisite selectivity for their cognate targets. With the exception of those with antibacterial/antifungal activity, all of the spider-venom peptides to be discussed in this review contain an ICK motif.

## 4. No Pain, Much Gain: Spider Toxins with Analgesic Potential

Normal nociceptive pain is a key adaptive response that limits our exposure to potentially damaging or life-threatening events. In contrast, aberrant long-lasting pain transforms this adaptive response into a debilitating and often poorly managed disease. About 20% of adults suffer from chronic pain, a figure that increases to 50% for those older than 65 [49]. In 2007, global sales of pain medications totaled $34 billion [50], highlighting the pervasive nature of this condition. Nevertheless, there are few drugs available for treatment of chronic pain, and many of these have limited efficacy and significant side-effects. Recently, a number of ion channels have been shown to be critical players in the pathophysiology of pain, and in many cases the most potent and selective blockers of these channels are spider-venom peptides. Here we review some of these peptides with promise as drug leads or as analgesics in their own right.

### 4.1. Modulators of Acid Sensing Ion Channels

Acid sensing ion channels (ASICs) are proton-gated sodium channels that open in response to low pH. They belong to the epithelial sodium channel/degenerin (ENaC/DEG) superfamily of ion channels which have the same overall topology and selectivity for transporting sodium [51]. However, ASICs are distinguished by their restriction to chordates, their predominantly neuronal distribution, and their activation by decreases in extracellular pH [52,53]. To date, seven ASIC subunits have been identified: ASIC1a, ASIC1b, and ASIC1b2 (splice isoforms from the ASIC1 gene), ASIC2a and ASIC2b (splice isoforms of the ASIC2 gene), ASIC3 and ASIC4. Functional ASIC channels comprise either homomeric or heteromeric trimers of these subunits. ASIC2b and ASIC4 are insensitive to protons and do not form homomeric channels, but rather are incorporated into heteromeric channels and may modify the kinetics of channel activation and inactivation. The different combinations of subunits allow the different trimeric channels to sense a wide range of extracellular pH changes.

ASIC1a is the most abundant ASIC subunit in the central nervous system (CNS) and it has the highest affinity for protons [53]. It has been implicated as a novel therapeutic target for a broad range of pathophysiological conditions including pain, ischemic stroke, depression, and autoimmune and neurodegenerative diseases such as multiple sclerosis, Huntington’s Disease, and Parkinson’s Disease [53,54,55,56]. Inhibitors of ASIC1a might therefore be therapeutically valuable for some of these conditions. The only potent and specific inhibitor of ASIC1a that has been identified to date is π-theraphotoxin-Pc1a (π-TRTX-Pc1a; also known as psalmotoxin-1 (PcTx1)), a 40-residue ICK peptide isolated from the venom of the Trinidad chevron tarantula *Psalmopoeus cambridgei.*π-TRTX-Pc1a inhibits homomeric ASIC1a channels, but not other ASIC subtypes, with an IC_50_ of 0.9 nM [30]. π-TRTX-Pc1a was shown to be an effective analgesic, comparable to morphine, in rat models of acute pain [57] and peripheral administration of this peptide resulted in neuroprotection in a mouse model of ischemic stroke even when administered hours after injury [58].

π-TRTX-Pc1a is only effective when administered intrathecally or by intracerebroventricular injection [57]. Thus, native π-TRTX-Pc1a is unlikely to be a clinically useful analgesic except in the most chronic pain sufferers as intrathecal administration is an invasive method of drug delivery with inherent risks [59]. As for Prialt®, a peptide from cone snail venom that was recently approved for the treatment of chronic pain [60], intrathecal π-TRTX-Pc1a use would likely be limited to management of severe chronic pain in patients who are intolerant or refractory to other treatments. Thus, there is much interest in developing mimetics of π-TRTX-Pc1a that might be orally active or at least deliverable via subcutaneous or intramuscular injection. Thus, several attempts have been made to model the π-TRTX-Pc1a:ASIC1a interaction [61,62] with a view to providing a template that can be used for *in silico* screening and/or rational design to develop small-molecule mimetics of π-TRTX-Pc1a. Thus, even in cases where a spider-venom peptide itself may not be a viable therapeutic, it can still be an invaluable tool for target validation and for providing a pharmacophore for rational drug design.

### 4.2. Modulators of Voltage-Gated Sodium Channels

Voltage-gated sodium (Na_V_) channels provide a current pathway for the rapid depolarization of excitable cells that is required to initiate an action potential. Functional channels are composed of a pore forming α subunit whose gating and kinetics is modified via association with one of four β subunits. The α subunits are classified into nine different subtypes, denoted Na_V_1.1 to Na_V_1.9 [63], and they are further characterized by their sensitivity to tetrodotoxin (TTX). Na_V_1.5, Na_V_1.8 and Na_V_1.9 are TTX-resistant whereas all other subtypes are TTX-sensitive.

Of the nine Na_V_ subtypes, Na_V_1.3, Na_V_1.7, and Na_V_1.8 are involved in pain signaling [64,65]. However, in recent years, Na_V_1.7 has emerged as perhaps the best validated pain target based on several remarkable human genetic studies. Gain-of-function mutations in the gene encoding the α subunit of Na_V_1.7 (*SCN9A*) underlie two painful neuropathies known as paroxysmal extreme pain disorder (PEPD) and inherited erythromelalgia (IE) [66,67], whereas loss-of-function mutations in *SCN9A* result in a *congenital indifference to all forms of pain* [68,69]. Remarkably, apart from their complete inability to sense pain, partial loss of smell (hyposmia) is the only other sensory impairment in individuals with this channelopathy [70]; they have no motor or autonomic dysfunction, with normal blood pressure and temperature regulation. Na_V_1.7 is located at the terminal of sensory neurons, where it is ideally positioned to serve its proposed role as a threshold channel that amplifies pain signals transmitted above a certain level [71].

The preferential expression of Na_V_1.7 in peripheral sensory and sympathetic neurons makes it an ideal target for novel analgesics. Indeed, it is probable that the known analgesic effects of a number of nonspecific Na_V_ channel blockers such as the local anaesthetic lidocaine, tricyclic antidepressants such as amitriptyline, and anticonvulsants such as carbamezepine are at least in part mediated through their effects on Na_V_1.7. However, the nonspecific block of Na_V_ channels by these drugs means that they are only efficacious at or near toxic levels, with numerous CNS-related side-effects such as dizziness and ataxia [64]. Thus, *subtype-specific* blockers of Na_V_1.7 are likely to be useful drugs for treatment of chronic pain as well as inherited neuropathies such as IE and PEPD [64,65,71]. Recent studies have revealed that spider venoms may provide an excellent source of such subtype-specific blockers.

Modulation of Na_V_ channels is a dominant pharmacology in spider venoms [72], indicating that spiders long ago evolved the capacity to block Na_V_ channels as a mechanism for killing insect prey. Since the insect Na_V_ channel shares 55–60% identity with each of the vertebrate Na_V_ subtypes [28] it is perhaps not surprising that numerous spider toxins have been isolated with activity against vertebrate Na_V_ channels. However, according to ArachnoServer [20,21], only three peptide toxins have been isolated thus far with activity against vertebrate Na_V_1.7 channel, and only six toxins in total have been isolated with submicromolar potency against Na_V_1.3, Na_V_1.7, or Na_V_1.8 (Table 1). All of these toxins were isolated from members of the Theraphosidae family (commonly known as tarantulas).

**Table 1:** Spider-venom peptides with submicromolar potency against Na_V_1.3, Na_V_1.7, or Na_V_1.8^1^.

Toxin Name	No. of Residues	ICK Scaffold	IC_50_ (nM) against Various Na_V_ Subtypes							
			1.1	1.2	1.3	1.4	1.5	1.6	1.7	1.8
β-TRTX-Tp1a	35	Yes	NA^2^	NA	NA	NA	NA	NA	51	27^3^
β-TRTX-Tp2a	30	Yes	NA	41	102	NA	79	26	0.3	146
β-TRTX-Ps1a	34	Yes	610	0.6	42	288	72	NA	NA	>1000
β-TRTX-Cm1a	33	Yes	523	3	NA	888	323	NA	NA	>1000
β-TRTX-Cm1b	33	Yes	407	8	88	400	1634	NA	NA	>2000
δ-TRTX-Cj1a^4^	33	Yes	NA	NA	NA	NA	32	130	130	NA

1. Data extracted from ArachnoServer (http://www.arachnoserver.org) on 01/11/10; 2. NA indicates data not available; 3.The Na_V_ subtype against which a toxin is most active is underlined; 4. This toxin does not block the channel but rather delays its inactivation.

The most potent known blocker of human Na_V_1.7, with an IC_50_ of 0.3 nM, is β-TRTX-Tp2a (Protoxin II), a 30-residue ICK peptide isolated from the venom of the Green velvet tarantula *Thrixopelma pruriens*. This toxin has a 100-fold selectivity for human Na_V_1.7 compared with Na_V_1.2, Na_V_1.3, Na_V_1.5, Na_V_1.6 and Na_V_1.8 [73,74]. Nevertheless, β-TRTX-Tp2a still has relatively high potency against Na_V_1.2 (IC_50_ = 41 nM) and Na_V_1.5 (IC_50_ = 79 nM) and consequently it is lethal to rats when injected intravenously at 1.0 mg/kg or by intrathecal administration at 0.1 mg/kg. In contrast, intrathecal administration of β-TRTX-Gr1b, a related toxin from the venom of the Chilean rose tarantula *Grammostola rosea* that is 89% identical to β-TRTX-Tp2a, induced analgesia in a variety of rat pain models without any confounding side-effects, and the peptide did not exhibit cross tolerance with morphine [75]. It is therefore likely that the Na_V_ subtype selectivity of β-TRTX-Gr1b, which remains to be determined, is very different to that of β-TRTX-Tp2a. Structure-function studies of closely related spider-venom peptides with different Na_V_ selectivity profiles, such as β-TRTX-Tp2a and β-TRTX-Gr1b, should provide structure-activity relationships that can be used to rationally design selective blockers of Na_V_1.7 with therapeutic potential as novel analgesics.

### 4.3. Modulators of P2X Receptors

P2X purinergic receptors are ATP-gated non-selective ion channels permeable to Na^+^, K^+^ and Ca^2+^ [76]. Currently, seven subunits (P2X_1-7_) are known [77] and functional P2X channels are formed by association of these subunits to form homomeric or heteromeric trimers [78]. To date, six different heteromeric channels with unique pharmacological properties have been discovered [77]. Of these, P2X_3_ [79], P2X_4_ [80], and P2X_7_ [77] are involved in a range of pain states. Several antagonists of these receptors have been patented and some P2X_7_ antagonists have already entered Phase I and II clinical trials [81].

P2X_3_ is the best-studied subtype with regards to pain. Consistent with its localization on ascending nociceptive sensory neurons [79], it has been found to be involved in acute pain, inflammatory pain, chronic neuropathic pain, visceral pain, migraine pain, and cancer pain [79,82]. Very few subtype-specific antagonists of P2X_3_ are known [83,84,85]. Recently, however, a potent and selective modulator of P2X_3_ was isolated from the venom of the central Asian spider *Geolycosa sp.* and named purotoxin-1 (PT1) [29]. PT1 is a 35-residue peptide with four disulfide bonds, three of which form an ICK motif. It has a complex pharmacology that leads to a concentration-dependent prolongation of channel desensitization, with an IC_50_ of ~12 nM [29].

The analgesic potential of PT1 was measured in rat models of acute and chronic inflammatory pain in which hind paw withdrawal latency was measured after induction of thermal hyperalgesia by injection of either carrageenan or Freund’s complete adjuvant, respectively. PT1 exhibited similar analgesic effects as the P2X_3_ antagonist A-317491, but the amount of PT1 required was three orders of magnitude lower [29]. PT1 was also effective in reducing the number of nocifensive events triggered by the injection of capsaicin or formalin [29]. Thus, PT1 appears to be a promising lead compound for the development of analgesics that target P2X_3_ receptors. PT1 is the first P2X modulator isolated from spider venoms and therefore it will be interesting in future studies to examine how widespread this pharmacology is in the venoms of these animals.

### 4.4 Spider-Venom Peptides that Modulate Other Pain Targets

In addition to the molecular targets discussed above, there are several other ion channels and receptors that have either been validated or are being actively investigated as analgesic targets. For example, Ziconitide (Prialt®), an analgesic peptide derived from cone snail venom, targets Ca_V_2.2 channels [86], while another cone snail peptide that targets the norepinephrine transporter [87] is currently in Phase II clinical trials for the treatment of post-operative pain. Several transient receptor potential (TRP) channels including TRPV1, TRPV4, TRPA1, and TRPM8 are also being investigated as analgesic targets and a number of TRPV1 antagonists are in clinical trials [88,89]. Spider venoms represent a potential source of modulators for all of these drug targets. For example, block of Ca_V_ channels is a dominant pharmacology in spider venoms [72]; more than 65 blockers of vertebrate Ca_V_ channels are currently listed in the ArachnoServer database, of which 17 are active on Ca_V_2.2. In addition, several spider-venom peptides have been isolated that modulate that activity of TRPV1 [32]. However, very few studies have explored whether spider-venom peptides are capable of targeting ligand-gated receptors, G-protein coupled receptors, or neurotransmitter transporters, and this is likely to be a fruitful line of enquiry for future research.

## 5. Antiarrhythmic Drugs from Spider Venoms

Mechanosensitive channels (MSCs), sometimes referred to as stretch-activated channels, are found in all cells [90], but mechanosensitivity is best viewed as a phenotype rather than a genotype [91]. Only two selective inhibitors of MSCs have been isolated, namely M-TRTX-Gr1a (GsMTx4) and κ-TRTX-Gr2a from the venom of the tarantula *Grammostola rosea* [92,93]; the latter peptide is identical to κ-TRTX-Ps1b (Phrixotoxin-2) isolated from venom of the Chilean copper tarantula *Paraphysa scrofa*. κ-TRTX-Gr2a/κ-TRTX-Ps1b is a low affinity blocker of MSCs, with a *K*_d_ of 6 μM in rat astrocytes, but it potently inhibits K_V_4.2 and K_V_4.3 channels, with IC_50_ values of 34 and 71 nM, respectively. In contrast, M-TRTX-Gr1a is a significantly more potent inhibitor of MSCs, with a *K*_d_ of 630 nM in rat astrocytes [92], and it has proved to be a valuable tool for study of MSCs [31].

Atrial fibrillation results from stretching of the atrial chamber, which has been associated with the activity of mechanosensitive channels. Block of these channels presumably explains the remarkable observation that M-TRTX-Gr1a suppresses atrial fibrillation in dilatated rabbit heart [94]. This suggests that MSCs might be a novel target for antiarrhythmic agents. M-TRTX-Gr1a itself is unlikely to be a useful therapeutic agent because of its unusual mode of action. It does not interact directly with MSCs, since an enantiomer comprised entirely of D-amino acids is equipotent with the native peptide [31]. Rather, the peptide perturbs the channel-bilayer boundary by partitioning into the membrane [95,96], and this membrane-disrupting activity presumably also underlies its antimicrobial activity [97]. Nevertheless, M-TRTX-Gr1a is likely to be a useful tool for determining the potential of MSCs as a therapeutic target for the treatment of pathologies as diverse as cardiac arrhythmias, spinal cord damage, muscular dystrophy, and gliomas [31].

## 6. Spider Toxins for Treating Erectile Dysfunction

Penile erection is a complex process initiated by activation of parasympathetic pelvic nerves, resulting in arterial dilatation followed by relaxation of corpora cavernosa [98]. Nitric oxide (NO) plays a major role in the generation and maintenance of intracavernous pressure and penile erection [99]. NO, which is released from nitrergic nerves within the trabecular and arterial tissues as well as by the endothelial tissue of penile arteries, exerts its relaxing action by activating soluble guanylyl cyclase. This causes an increase in intracellular cGMP which relaxes the smooth muscles of the cavernous body and results in penile erection.

There are several drugs on the market today for treatment of erectile dysfuncion (ED), including sildenafil (Viagra®), tadalafil (Cialis®), and vardenafil (Levitra®) [100]. These drugs all affect phosphodiesterase type 5 (PDE5), which is present in large amounts in the penis. Inhibition of PDE5 leads to increased levels of cGMP and hence increased blood flow to the penis [101]. The aforementioned PDE5-blocking drugs have similar side effects including headache, flushing, dyspepsia, nasal congestion, impaired vision, photophobia and blurred vision [102]. Hence, there is a need for better drugs with fewer side effects for the treatment of ED.

In South America, humans bitten by the “armed-spider” *Phoneutria nigriventer* experience a variety of symptoms including priapism. The toxin responsible for this effect, δ-ctenitoxin-Pn2a (δ-CNTX-Pn2a; Tx2-6) was isolated in 1992 [103] and subsequently found to modulate the activity of Na_V_ channels [104]. δ-CNTX-Pn2a is a 48-residue toxin with five disulfide bonds; it is unclear whether it contains an ICK motif. The toxin has a complex pharmacology that results in inhibition of Na_V_ channel inactivation and a hyperpolarizing shift in the channel activation potential [104]. *Intracerebroventricular* injection of δ-CNTX-Pn2a into mice results in scratching, hypersalivation, lachrymation, sweating, and agitation followed by spastic paralysis of the anterior and posterior extremities and death [103]. However, *subcutaneous* injection of δ-CNTX-Pn2a into rats induced erection, and rats with severely depressed erectile function could be normalized by subcutaneous administration of δ-CNTX-Pn2a [105]. A minimum dose of only 0.006 μg/kg was required to cause an erection in mice when injected directly into the corpus cavernosum; at this dose, no local and systemic collateral toxic effects were observed and the erection was lost after 120–140 min [106].

The potency and specificity of δ-CNTX-Pn2a makes it an attractive lead molecule for the development of new therapeutics for ED treatment that might have fewer side effects than current drugs. The pharmacology inherent in δ-CNTX-Pn2a may be widespread in spider venoms, at least amongst members of the Ctenidae family (which currently comprises 475 species [107]), since orthologous toxins are found in several other *Phoneutria* species as well as a related ctenid spider (see ArachnoServer entry http://www.arachnoserver.org/toxincard.html?id=16).

## 7. Antibacterial and Antifungal Toxins

The introduction of antibiotics in the 1930s and 1940s was in large part responsible for the dramatic decline in the mortality rate from communicable diseases in developed countries [108]. However, bacteria are remarkably proficient at adapting to environmental stresses, and they have evolved at least one mechanism of resistance for all 17 classes of antibiotics that have been developed to date [109]. The recent widespread emergence of antibiotic resistance in clinically important bacterial pathogens such as *Staphylococcus aureus*, *Streptococcus pneumoniae*, and *Enterococcus faecalis*, combined with a dramatic decrease in the rate of development of new antibiotics, has led some to suggest that we may be approaching the post-antibiotic era [109]. While this may overstate the problem, there is nevertheless an urgent need to develop new antimicrobials with novel mechanisms of action.

The recent successful introduction of the lipopeptide antibiotic daptomycin [110] has rekindled interest in antimicrobial peptides [111]. To date, 40 membrane-acting antimicrobial peptides (MAMPs) have been isolated from the venom of four different families of araneomorphs, suggesting that antimicrobial activity is widespread in this infraorder of spiders. These MAMPs often have a wide range of antimicrobial activities, with some toxins active against Gram-positive and Gram-negative bacteria as well as fungal pathogens such as *Candida albicans* [112]. Some MAMPs also have anti-trypanosomal activity [113]. Curiously, no MAMPs have thus far been isolated from the venom of mygalomorph spiders. It is possible that MAMPs were recruited into araneomorph venoms following the split from mygalomorphs around 280 million years ago [114]. 

MAMPs differ from most other spider-venom peptides in their structure and mode of action. Rather than utilizing the ICK fold common to most spider toxins, MAMPs are α-helical amphipathic peptides that interact with and perturb cell membranes to yield their antimicrobial effects [115,116,117,118]. This mode of action can be potentially problematic from a therapeutic perspective since MAMPS that interact nonspecifically with cell membranes are cytolytic [119]. Indeed, this property is likely to be the basis of their biological function in spider venoms. Although it has been proposed that MAMPS might protect the spider’s venom apparatus against infection [115] their primary role is more likely to be as membrane disrupting agents that augment the activity of the disulfide-rich neurotoxic peptides by facilitating their spread [120,121].

It has been shown that the cytolytic activity of at least some spider-venom MAMPs can be minimized by truncation without significantly disrupting their antimicrobial activity [122]. Nevertheless, the therapeutic use of these peptides is likely to be limited by their inherent susceptibility to proteolysis, which is likely to result in short gut and plasma half-lives. Whether this problem can be solved by strategies such as cyclization or grafting key sequence elements onto more stable ICK scaffolds [123] remains to be seen.

## 8. Antimalarial Toxins

The antimicrobial action of spider toxins is not limited to bacteria and fungi, but also extends to the malaria parasite. There were 243 million cases of malaria in 2008, resulting in a death every 35 seconds, and most of these were children under the age of five [124]. Malaria is caused by *Plasmodium* infections spread by female anopheline mosquitoes. There are five genera of *Plasmodium* that cause malaria, with *Plasmodium falciparum* being the most virulent. Widespread resistance to chloroquine has made this drug largely ineffective for treating *Plasmodium falciparum* in high-transmission areas and few cheap alternatives are available [125].

U_1_-TRTX-Pc1a (Psalmopeotoxin I) and U_2_-TRTX-Pc1a (Psalmopeotoxin II) are ICK peptides isolated from the venom of the Trinidad chevron tarantula *Psalmopoeus cambridgei* that are effective against the intra-erythrocyte stage of *Plasmodium falciparum*. Interestingly, this is the same spider from which π-TRTX-Pc1a, the most potent known blocker of ASIC1a, was isolated, indicating that a single spider can provide multiple therapeutic leads. U_1_-TRTX-Pc1a and U_2_-TRTX-Pc1a are unrelated peptides that comprise 33 and 28 residues, respectively. They inhibit intra-erythrocyte development of *Plasmodium falciparum* with ED_50_ values of 1.1–1.6 μM but, unlike most MAMPS, they do not have hemolytic, antibacterial or antifungal activity. The mode of action of these peptides is unknown. It seems unlikely that they directly target the malaria parasite since this would require the peptides to traverse both the erythrocyte membrane as well as the parasitophorous vacuolar membrane that encapsulates the parasite. One possibility is that these toxins target the new permeability pathways that are established in the erythrocyte membrane following parasite invasion [126]. Thus, in addition to being useful therapeutic leads, these peptide toxins might help validate a new anti-malarial drug target.

## 9. Discussion

**Figure 2:**
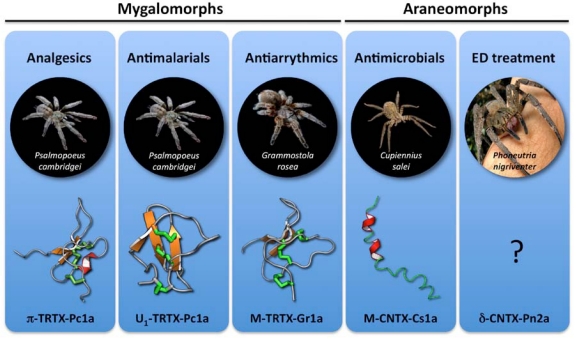
Spider-venom peptides that are serving as therapeutic leads. A photo of the spider from which each toxin was isolated is shown, and the name and 3D structure of the toxin is indicated at the bottom of each panel. Disulfide bonds are shown as green tubes, while β strands and α helices are highlighted in orange and red, respectively. The structure of δ-CNTX-Pn2a has not yet been determined. Note that therapeutic leads have been isolated from both “modern” (araneomorph) and “primitive” (mygalomorph) spiders [3].

In the preceding sections we surveyed a number of spider-venom peptides with potential therapeutic applications (Figure 2). With the exception of the MAMPs that have antimicrobial/antifungal activity, all of these peptides are stabilized by an inhibitor cystine knot motif. Thus, they are unlikely to suffer from one of the major problems typically associated with peptide drugs, namely rapid proteolytic degradation. As any surviving bite victim will attest, the devastating *in vivo* efficacy of the 42-residue lethal Na_V_ channel toxin from the Australian funnel-web spider [42,127] plainly demonstrates how minute quantities of ICK peptides can be effective in humans. Should rapid proteolysis prove to be an issue for a spider-venom peptide of therapeutic interest, strategies such as D-amino acid substitution of susceptible residues, cyclization to reduce conformational flexibility, and protection of the termini via C-terminal amidation or use of N-terminal pyroglutamate could be employed to improve proteolytic resistance [37,128,129]. Indeed, spiders routinely employ C-terminal amidation as a protection strategy, with ~12% of all known spider toxins containing this posttranslational modification [21].

Because of their inherent proteolytic resistance, the plasma half-life of ICK peptides is likely to be determined by the rate at which they are cleared by glomerular filtration, which efficiently removes small proteins and peptides that are not bound to carrier proteins such as serum albumin [130]. However, there are a variety of strategies that can be employed to reduce peptide clearance rates, such as increasing the peptide mass by PEGylation, conjugation to carrier proteins, or by making peptides more hydrophobic in order to enhance their association with serum albumin; the latter approach can also cause hydrophobic depoting, which provides sustained release from a subcutaneous injection [37]. 

For acute life-threatening conditions as well as chronic conditions such as persistent pain, subcutaneous injection is likely to be an acceptable route of peptide-drug administration. In certain pathologies where quality of life is dramatically reduced, intrathecal administration may even be a viable option if the difficulties associated with peptide delivery are outweighed by the benefits of treatment. Generally, however, oral delivery is likely to be desirable. The intrinsic stability of ICK peptides is likely to facilitate the development of oral delivery strategies since they will presumably have much longer gut and plasma residence times than typical peptides. Moreover, spider-venom ICK peptides are small enough to consider alternative routes of administration such as intranasal, transdermal, and pulmonary [131].

An alternative but complementary approach is to develop small-molecule mimetics of spider-venom peptides. The epitope (pharmacophore) that mediates the interaction of these peptides with their cognate receptors or ion channels can be remarkably small. For example, the interaction between the spider-venom peptide ω-hexatoxin-Hv1a and invertebrate Ca_V_ channels is mediated by a pharmacophore comprising only three spatially contiguous residues with a solvent-accessible surface area of ~200 Å [132,133], which approximates the typical solvent-accessible surface area of a small drug [134]. As long as a high-quality structure of the peptide is available, this enables *ab initio* design of nonpeptide mimetics [135,136], identification of small molecule mimetics via *in silico* screening of chemical libraries, or a combination of these approaches.

## 10. Conclusions

Over a period of more than 300 million years, spiders have evolved an extensive library of bioactive peptides. Moreover, in contrast with man-made combinatorial peptide libraries, spider-venom peptides have been pre-optimized for high affinity and selectivity against a diverse range of molecular targets. It is therefore not surprising that numerous spider-venom peptides have been characterized that potently and selectively modulate the activity of a diverse range of therapeutic targets. These include peptides that target Na_V_ channels, ASICs, MSCs, and purinergic receptors as well as peptides with antimalarial and antimicrobial activity. Most of these peptides contain an ICK motif, and their extraordinary stability provides a variety of delivery options for therapeutic administration. Only a small fraction of spider-venom peptides have been characterized, and continued technical advances in the venoms-based drug discovery process are likely to uncover many new therapeutic leads from spider venoms.
